# Multiple cerebral infarction diagnosed as Eosinophilic Granulomatosis with Polyangiitis by autopsy

**DOI:** 10.1186/s12883-019-1515-z

**Published:** 2019-11-15

**Authors:** Kenichiro Hira, Hideki Shimura, Riyu Kamata, Masashi Takanashi, Akane Hashizume, Keiji Takahashi, Mizuho Sugiyama, Hiroshi Izumi, Nobutaka Hattori, Takao Urabe

**Affiliations:** 1 Department of Neurology, Juntendo University Urayasu Hospital, 2-1-1 Tomioka, Urayasu, Chiba, 279-0021 Japan; 2 Department of Neurology, Juntendo University Koshigaya Hospital, 560 Fukuroyama, Koshigaya, Saitama, 343-0032 Japan; 3 Department of Pathology, Juntendo University Urayasu Hospital, 2-1-1 Tomioka, Urayasu, Chiba, 279-0021 Japan; 4 Department of Neurology, Juntendo University School of Medicine, 2-1-1 Hongo, Bunkyo-ku, Tokyo, 113-8421 Japan

**Keywords:** Eosinophilic granulomatosis with polyangiitis (EGPA), Multiple cerebral infarction, Hypereosinophilia, Intracardiac thrombus

## Abstract

**Background:**

Eosinophilic granulomatosis with polyangiitis (EGPA) is a rare systemic vasculitis of unknown cause involving the brain and accompanied by prominent eosinophilia. Intracardiac thrombosis is a major cardiac complication of EGPA that may cause thromboembolism.

**Case presentation:**

A 53-year-old man presenting with abulia (consciousness disturbance) and left upper limb paralysis was admitted to our hospital. His case was complicated by penetrating branches, small vessel infarcts, and endocardial thrombosis in the right and left ventricle. Cardiomyopathy was also observed. Sixteen days after admission, the patient died from intracranial hemorrhage. Brain autopsy revealed two major findings: 1) large hemorrhagic infarction caused by cardiac embolism; and 2) granuloma and eosinophil infiltration. Vasculitis was accompanied by eosinophil infiltration in the cortical blood vessels and granuloma.

**Conclusions:**

In this case study, we report autopsy findings of brain infarction in a patient with EGPA and endocardial thrombosis. The brain infarction was caused by the cardiac embolisms and vasculitis.

## Background

Eosinophilic granulomatosis with polyangiitis (EGPA) causes bronchial asthma and allergic rhinitis as a preceding symptom and results in vasculitis accompanied with eosinophilia [1, 2]. In rare cases, EGPA is thought to exhibit multiple cerebral infarction in cortical, subcortical, and watershed areas, and mechanisms of vasculitis and special granules secreted by eosinophils have been considered [3]. Furthermore, because of the presence of left ventricular thrombus due to eosinophilic myocarditis, embolism is considered a cause, but the details are unknown [3].

Here, we describe pathological findings showing brain infarction caused by both cardiac embolism and eosinophilic vasculitis in a patient with EPGA.

## Case presentation

A 53-year-old man, with no medical history, was diagnosed with influenzae virus infection and was administered oseltamivir phosphate 2 weeks before developing ischemic stroke. There was no prior bronchial asthma or allergic rhinitis. There were no symptoms such as weight loss, polyneuropathy, myalgia, arthralgia, or gastrointestinal bleeding. There were no abnormalities in blood collection as a result of the physical examination. He was found by a neighbor at home in a left lateral decubitus position and was taken to our hospital. He presented with abulia and left upper limb paralysis. He was in a state of collapse for several days, and there were decubituses in the left upper and lower limbs. In addition, he showed disseminated intravascular coagulation and was in a septic state. Vital signs showed mild fever (body temperature: 37.7 °C), but blood pressure, pulse rate, respiratory rate, and arterial oxygen saturation by pulse oxymetry were within normal ranges. Chest X-ray and computed tomographic imaging of the chest showed no abnormality. An electrocardiogram showed ST depression in chest leads indicating either ischemic change along the entire circumference of the heart or cardiomyopathy. Laboratory findings showed no leukocytosis (8900 /μl; normal range, 4000–8000 /μl), but hypereosinophilia (1020 /μl; normal range, 0–400 /μl) and an elevated IgE (9372 IU/ml; normal range, 18–501 IU/ml). Elevated CRP (6.4 mg/dl; normal range, 0–0.3 mg/dl) was observed. Prothrombin time international normalized ratio was 1.37, activated partial thromboplastin time was 30.5 s, D-dimer was 37.8 μg/dl (normal range, 0–1 μg/dl), fibrinogen degradation products was 79.9 μg/dl (normal range, 0–5 μg/dl), fibrinogen was 261 mg/dl (normal range, 150–400 mg/dl), and soluble fibrin was 114.4 μg/dl (normal range, 0–7 μg/dl). Protein S total level was 57% (normal range, 60–150%) and activity was 28% (normal range, 73.7–146.3%). Protein C was within normal range. Myeloperoxidase-antineutrophil cytoplasmic antibodies and cytoplasmic ANCA were negative. Viruses and fungi that cause myocarditis, such as human immunodeficiency virus, adenoviruses, group A and B coxsackieviruses, Cytomegalovirus, echovirus, Epstein-Barr virus, influenza A and B viruses, Candida antigens, and Aspergillus antigens were all negative by blood tests. A bone marrow biopsy was also performed at autopsy. There was no clonality on flow cytometry. The FIP1L1–PDGFRA fusion gene (4q12) was not detected by fluorescence in situ hybridization. Bone marrow findings showed no hematologic malignancies causing hypereosinophilia.

**Fig. 1:**
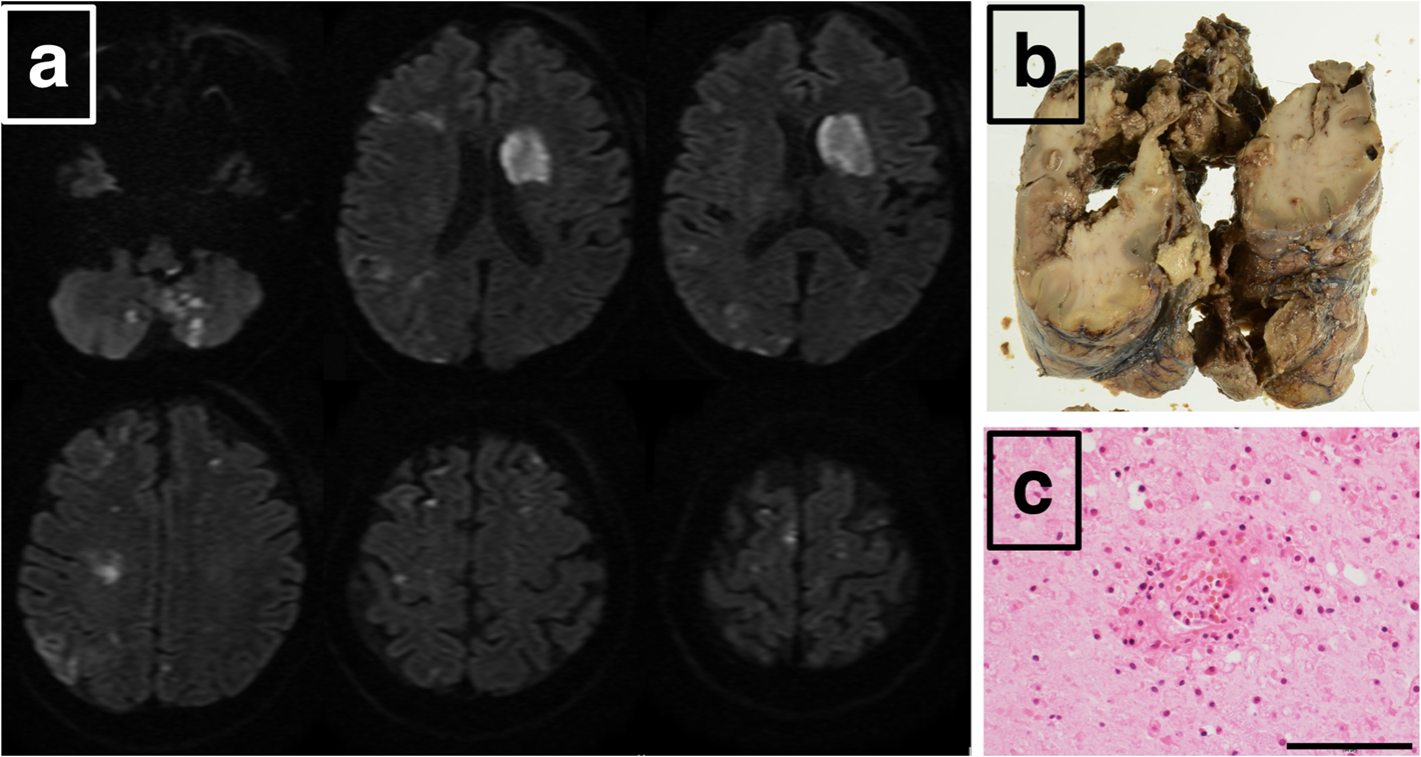
Diffusion Weighted Magnetic Resonance Imaging of cerebrum (**a**) / brain autopsy findings (**b**) / Eosinophilic granulomatosis in brain (**c**). On admission, there were multiple infarctions which had a mixture of small infarction and large hemorrhagic infarction. Multiple small cerebral infarct lesions in the cortex and subcortex were caused by vasculitis and eosinophil infiltration

Magnetic resonance imaging of the brain revealed ischemic infarcts in the left caudate nuclei, bilateral cortex, subcortical region, bilateral watershed area, brain stem, and cerebellum (Fig. 1a). No obvious blood vessel obstruction or stenosis was observed by magnetic resonance angiography. Transthoracic echocardiography showed intramural thrombus with left ventricle and right ventricle involvement. Ejection fraction and wall motion were within normal ranges, and there were no valvular disease. In carotid ultrasonography, hypertrophy of the intima-media thickness and blood flow abnormality were not observed.

**Fig. 2:**
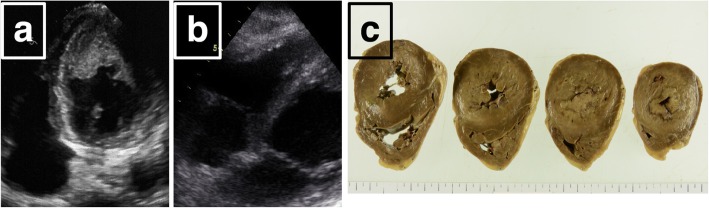
Intramural thrombus in left (**a**) and right ventricle (**b**) detected by transthoracic echocardiography. / Formalin-fixation of left ventricle (**c**). Both ventricular chambers were narrowed by the thrombus. Scale bar: 100µm

The patient was diagnosed as reactive hypereosinophilic syndrome (HES_R_). He was administered 200 mg/day aspirin for acute cerebral infarction. Consciousness disturbance began to improve. Since we confirmed intracardiac thrombus by cardiac ultrasound on hospital day 5 (Fig. 2a, b), he was administered 14,800 units of unfractionated heparin for 24 h. On hospital day 6, his consciousness level worsened to coma caused by cerebral hemorrhage. We performed a burr-hole evacuation, but, on hospital day 16, he died.

Autopsy was conducted with the family’s consent. Autopsy findings revealed the following: 1) granulomas with epithelial cells and eosinophil infiltration; and 2) vasculitis accompanied by fibrinoid necrosis in the kidney, lung, colon, gallbladder, stomach, liver, spleen, heart, and pancreas. In the left ventricle of the heart, thickening of the wall, narrowing of the lumen, and thrombosis was observed around the circumference (Fig. 2c). Thrombus was also observed in the right ventricle (Fig. 2c). Granuloma formation was present in the blood vessels of the right coronary artery and, in the left ventricular myocardium, myocardial cell shedding, inflammatory cell infiltration such as macrophages and lymphocytes, and partial fibrosis were observed. The brain was severely damaged by infarction with hemorrhage (Fig. 1b). In addition to thromboembolic infarction, we observed vasculitis accompanied by eosinophil infiltration in the parenchymal small arteries. The autopsy findings indicated that the patient had systemic eosinophilic polyangiitis that included the brain (Fig. 1c). Pathological study revealed that the cause of the cerebral infarction was the coexistence of cardiogenic cerebral embolism and cerebral vasculitis with eosinophilic infiltration by EGPA.

## Discussion and conclusions

EGPA is a vasculitis syndrome also known as allergic granulomatous angiitis or Churg Strauss syndrome. EGPA is a part of HES_R_, in which asthma and allergic rhinitis appear and symptoms due to vasculitis develop [1]. Symptoms of EGPA are peripheral neuritis, purpura, gastrointestinal ulcer, cerebral infarction, cerebral hemorrhage, and epicarditis [2]. The annual incidence of EGPA is approximately 2.7–3.4/million [4]. EGPA treatment includes prednisolone, immunosuppressants, and gamma globulin [5]. The prognosis in approximately 90% of patients is remission within 6 months [6]. Our patient did not have asthma, neuropathy, or other clinical symptoms before the onset of stroke. The 5-year survival rate of EGPA may be as high as 70%, but advanced age (≥65 years old), myocardial disorder, impaired renal function (Cr > 1.58 mg/dl), and severe gastrointestinal ischemia are detrimental prognostic factors [7]. In particular, myocardial injury is correlated with a high risk of death [8]. Approximately 50% of EGPA cases are ANCA positive [8]. In positive cases, the recurrence rate tends to be high and the death rate tends to be low, while, in negative cases, the recurrence rate is low (8.7%) and the mortality rate tends to be high [8]. Compared to ANCA-positive patients, ANCA-negative patients show cardiomyopathy more frequently, and their mortality rate is higher. On the other hand, the risk of relapse may be lower. In addition, ANCA positive patients tend to have more clinical vasculitis manifestations, such as peripheral neuropathy or renal disfunction [8]. While the difference between ANCA-negative EGPA and HES, the therapeutic effect is reported to be better and faster with EGPA than with HES [8]. About 50–60% of people diagnosed with HES are reported to have myocarditis [9], and about 35–65% of patients diagnosed with HES have neurological complications [10, 11]. Therefore, if eosinophils are increased in patients with myocarditis and there are no neurological complications, ANCA-negative EGPA should be considered.

Cerebral infarction occurs in 3–10% of EGPA cases [12]. A mechanism of cerebral infarction is that proteins, such as major basic protein and eosinophil peroxides, secreted from eosinophils activated by IL-5 impair nerve tissue and local thrombosis results from tissue factor in secretory granules [13, 14]. In addition, eosinophilia may cause microthrombosis by increasing procoagulant activity or increasing blood viscosity and decreasing the clearance of microthrombus [13, 14]. Cardiogenic embolism is the most likely cause of cerebral infarction in the watershed area. A total of 16.4% of patients with EGPA have myocarditis, but the occurrence of intracardiac thrombosis is rare [8]. Our literature search revealed only 24 cases of EGPA developing cerebral infarction, of which, 8 cases had myocarditis and 3 cases had intracardiac thrombosis. All of those cases were diagnosed as EGPA at the time of arrival at hospital. Interestingly, our patient developed cerebral infarction without allergic symptoms or asthma. For the diagnosis of EGPA, it is important to clarify the presence of microvascular granulomatosis or fibrinoid necrotizing vasculitis with marked eosinophil infiltration by tissue biopsy. However, in this case, tissue biopsy could not be performed due to exacerbation of the patient’s general condition, and granulomas and vasculitis with eosinophil infiltration of the whole body organs was confirmed by post mortem examination. In this case, the patients had ventricular thrombus as well as hypereosinophilia. We could not use high dose steroids, since high dose steroids might worse thrombus.

In this study, we report the pathological determination of cerebral infarction in a patient with EGPA. Pathological study revealed that the mechanism of cerebral infarction included multiple factors of vasculitis, eosinophil infiltration, and embolism caused by intracardiac thrombus. Thus, we speculate that 1) the watershed area and multiple cerebral infarct lesions in the cortex and subcortex were caused by vasculitis and eosinophil infiltration, while 2) the relatively large infarction lesions (Caudate nucleus) and penetration of the infarcts found in the branch area were caused by cardiac embolism.

We propose that myocarditis and intracardiac thrombus should be investigated when a large infarct lesion is observed in EPGA patients. Since pathological findings revealed that large infarction was caused by cardiac embolism and small infarction was caused by vasculitis, EGPA should be considered in cases of multiple cerebral infarction and increased eosinophils.

## Data Availability

All the data supporting our findings are provided within the manuscript.

## Abbreviation

EGPA: Eosinophilic granulomatosis with polyangiitis
